# Adipose-derived mesenchymal stromal cells modulate tendon fibroblast responses to macrophage-induced inflammation *in vitro*

**DOI:** 10.1186/s13287-015-0059-4

**Published:** 2015-04-16

**Authors:** Cionne N Manning, Catherine Martel, Shelly E Sakiyama-Elbert, Matthew J Silva, Shivam Shah, Richard H Gelberman, Stavros Thomopoulos

**Affiliations:** Department of Orthopedic Surgery, Washington University, 425 S Euclid, Box 8233, St Louis, MO 63110 USA; Department of Pathology and Immunology, Washington University, 425 S Euclid, Box 8233, St Louis, MO 63110 USA; Current address: Montreal Heart Institute, Université de Montréal, Montréal, Quebec Canada; Department of Biomedical Engineering, Washington University, One Brookings Drive, Campus Box 1097, St Louis, MO USA

## Abstract

**Introduction:**

Macrophage-driven inflammation is a key feature of the early period following tendon repair, but excessive inflammation has been associated with poor clinical outcomes. Modulation of the inflammatory environment using molecular or cellular treatments may provide a means to enhance tendon healing.

**Methods:**

To examine the effect of pro-inflammatory cytokines secreted by macrophages on tendon fibroblasts (TF), we established *in vitro* models of cytokine and macrophage-induced inflammation. Gene expression, protein expression, and cell viability assays were used to examine TF responses. In an effort to reduce the negative effects of inflammatory cytokines on TFs, adipose-derived mesenchymal stromal cells (ASCs) were incorporated into the model and their ability to modulate inflammation was investigated.

**Results:**

The inflammatory cytokine interleukin 1 beta (IL-1β) and macrophages of varying phenotypes induced up-regulation of pro-inflammatory factors and matrix degradation factors and down-regulation of factors related to extracellular matrix formation by TFs in culture. ASCs did not suppress these presumably negative effects induced by IL-1β. However, ASC co-culture with M1 (pro-inflammatory) macrophages successfully suppressed the effects of M1 macrophages on TFs by inducing a phenotypic switch from a pro-inflammatory macrophage phenotype to an anti-inflammatory macrophage phenotype, thus resulting in exposure of TFs to lower levels of pro-inflammatory cytokines (e.g., IL-1β, tumor necrosis factor alpha (TNFα)).

**Conclusions:**

These findings suggest that IL-1β and M1 macrophages are detrimental to tendon healing and that ASC-mediated modulation of the post-operative inflammatory response may be beneficial for tendon healing.

**Electronic supplementary material:**

The online version of this article (doi:10.1186/s13287-015-0059-4) contains supplementary material, which is available to authorized users.

## Introduction

Despite advances in operative techniques and rehabilitation methods, the outcomes of treatment of tendon and tendon-to-bone repair are highly variable, resulting in a substantial clinical burden [1,2]. An extraordinarily high rate of recurrent tear (as high as 94% for some patient populations) has been noted following rotator cuff tendon-to-bone repair [1,2]. Similarly, intrasynovial flexor tendon repair has been shown to be susceptible to gapping and rupture during the first three post-operative weeks, and to the formation of adhesions between the tendon and its sheath [3-8].

Following tendon repair, healing progresses through three overlapping phases: inflammation (days 1 to 7), proliferation (days 3 to 14), and remodeling (day 10 onward). Prior attempts to improve the repair process have largely targeted the later stages of healing and have had limited success [8-10]. Recent evidence suggests that fine modulation of inflammation in the earliest stages following surgical repair may be required for improved outcomes [11-14]. Although low levels of inflammatory cytokines are likely necessary to attract fibroblasts to the repair site [15,16], excessive inflammation after tendon repair has been identified as a key factor leading to poor clinical outcomes [11,13,14,17]. Macrophages are broadly classified into the classically activated M1 phenotype or the alternatively activated M2 phenotype. As pro-inflammatory M1 macrophages have been implicated in the poor healing response of tendons, depletion of these cells or blockade of pro-inflammatory factors using pharmaceutical and rehabilitation methods may improve healing [11,12,18]. *In vitro* studies of tendon fibroblasts (TFs) have shown significant up-regulation of matrix degradation and inflammation-related factors and down-regulation of extracellular matrix when treated with inflammatory factors such as IL-1β [19-22]. Taken together, these studies suggest that macrophages and, specifically, the M1 (pro-inflammatory) phenotype, may impede tendon healing due to their production of high levels of pro-inflammatory cytokines [11,12,18].

Prior *in vivo* and *in vitro* studies have suggested that the application of mesenchymal stromal cells (MSCs), by virtue of their ability to modulate the inflammatory environment, may improve the healing response [23-31]. The mechanisms by which MSCs regulate inflammation for improved wound healing remain unclear. MSCs may produce factors that directly protect fibroblasts from harmful cytokines, such as IL-1β. Alternatively, MSCs may modulate the innate immune response by promoting the differentiation of monocytes into anti-inflammatory macrophages (that is, M2 macrophages) as opposed to classically activated, pro-inflammatory macrophages (that is, M1 macrophages) [29-31]. These studies suggest that M1 macrophages and cytokines, such as IL-1β, are detrimental to tendon healing and controlling inflammation during healing using MSCs may lead to improved outcomes. The purpose of this study was to investigate the effects of macrophages on TFs and to examine the ability of adipose-derived mesenchymal stromal cells (ASCs) to modulate those effects *in vitro*. We hypothesized that IL-1β and M1 macrophages would negatively affect TFs (as evidenced by increased production of inflammatory factors and matrix metalloproteinases and decreased production of extracellular matrix) and that ASCs would attenuate this effect by modulating macrophage phenotype.

## Methods

### Overview

*In vitro* studies were performed to examine TF responses to inflammatory environments and the subsequent effect of ASCs. Specifically, TFs were cultured: (1) with and without IL-1β and (in transwell plates); and co-cultured (2) with ASCs with and without IL-1β; (3) with macrophages; or (4) with macrophages and ASCs (TFs were in one well, macrophages and ASCs were directly cultured in the transwell) (Additional file 1: Figure S1).

### Cell isolations and culture

TFs, ASCs, and macrophages were isolated from six-week-old male C57BL/6 J mice (Jackson Laboratories, Bar Harbor, Maine, USA). Tissues were dissected post mortem from normal animals allocated to other studies. TFs were isolated from tail tendon and ASCs were isolated from abdominal adipose tissue. The tissues were minced and digested in 0.2% collagenase Type IA. The digested tissue was collected by centrifugation and incubated in alpha-modified Eagle’s medium (Alpha-MEM) with 10% fetal bovine serum (FBS) and 1% penicillin/streptomycin (P/S). ASCs were selected by adherence and verified by flow cytometry (see below for flow cytometry methods). TFs and ASCs were used on passage 2 to 4. Macrophages were derived from total bone marrow using L929 cell-conditioned medium as a source of macrophage colony stimulating factor. Total bone marrow was obtained by flushing the femurs and tibiae of the mice with Roswell Park Memorial Institute (RPMI) medium-1640. The red blood cells were lysed and the resultant cell population was cultured in L929-conditioned medium (30% L929 supernatant + 10% FBS + 1% P/S in RPMI medium) for seven days. The macrophages were then primed to become either M1 or M2 macrophages or left untreated (M0). M1 priming consisted of 50 ng/ml IFNγ (R&D Systems, Minneapolis, MN, USA) and 100 ng/ml *Escherichia coli* lipopolysaccharide (LPS) (Invivogen, San Diego, CA, USA) in RPMI complete medium (RPMI + 10% FBS + 1% P/S) for 24 hours. M2 priming consisted of 10 ng/ml IL-4 (R&D Systems) in RPMI complete medium for 24 hours. Untreated macrophages (M0) were given fresh RPMI complete medium for 24 hours. All experiments were performed in duplicate.

### Characterization of macrophage phenotype

To verify the phenotype of the primed macrophages, the macrophage populations were analyzed by flow cytometry for cell surface markers and medium was analyzed for secreted cytokines. Surface markers for all macrophages (CD11b: eBiosciences clone M1/70, San Diego, CA, USA; and F480 antibody: eBiosciences clone BM8) and specifically for M2 macrophages (CD206: APC, AbD Serotec, Oxford, UK; and CD301: Alexa Fluor® 488, AbD Serotec) were assessed. Data were acquired on a BD FACS Canto Flow Cytometer (BD Biosciences, San Jose, CA, USA) and analyzed with FlowJo software (Treestar, Ashland, OR, USA). The gating strategy was devised to exclude cell debris and doublet cells by forward and side scatter (FSC and SSC, respectively). The cells of interest stained positive for both F480 and CD11b (general macrophage markers). The expression of M2 macrophage-specific markers (CD206 and CD301) in the selected macrophage population was then assessed. To further examine the macrophage phenotypes, the expression of pro-inflammatory cytokines in the cell supernatants was examined. After 24 hours of macrophage priming, the priming medium was replaced with fresh RPMI complete medium and the cells were cultured for an additional 24 hours prior to supernatant collection. Supernatant samples were stored at −80°C until analysis. The levels of IL-1β, TNFα, nitric oxide (NO), and prostaglandin E2 (PGE2) were assessed using commercially available kits (1: IL-1β ELISA, R&D Systems; 2: TNFα ELISA, R&D Systems,; 3: Nitric Oxide Colorimetric Assay, EMD Millipore Chemicals, Darmstadt, Germany; and 4: Prostaglandin E2 Parameter Assay Kit, R&D Systems). Statistical significance for flow cytometry and protein expression analyses was assessed using an analysis of variance (ANOVA) (for the effects of macrophage type) followed by a Fisher’s *post-hoc* test.

### Characterization of ASCs

ASCs were characterized via quantification of surface marker expression. The cells were dislodged by trypsin-ethylenediaminetetraacetic acid (EDTA) and stained with antibodies known to be expressed by MSCs (CD44: eBioscience clone IM7, San Diego, CA, USA; CD29: eBioscience clone HMb1-1) and hematopoietic cells (CD34: eBioscience clone RAM34; CD14: eBioscience clone Sa2-8) for 30 minutes at 4°C. Unstained cells served as a negative control. Data were acquired on a BD FACS Canto Flow Cytometer (BD Biosciences) and analyzed with FlowJo software (Treestar). The cells were gated based on their forward and side scatter properties to exclude debris and doublets. A fuller characterization of these cells as well as a colony-forming unit fibrolasts (CFU-F) assay was performed and reported in a separate publication to verify ASC characteristics [32]. Cells of interest stained positive for both F480 and CD11b (general macrophage markers).

### IL-1β-induced inflammation

To determine the effects of IL-1β on TFs, TFs were plated at 5.2 x 10^4^ cells/cm^2^ in six-well plates and treated with varying amounts of IL-1β (0 ng/ml, 0.01 ng/ml, 0.1 ng/ml, 1 ng/ml, 10 ng/ml, and 100 ng/ml; rat-derived, R&D Systems). The cells were cultured for one, two, and three days before the cell supernatant was collected and RNA was isolated.

### Macrophage-induced inflammation

To examine the effect of pro-inflammatory cytokines secreted by macrophages on TFs, an *in vitro* model was established of inflammation induced by macrophages. Macrophages of varying phenotypes (M0, non-polarized; M1, classically activated/pro-inflammatory; or M2, alternatively activated/anti-inflammatory) were co-cultured with TFs for one day using a transwell co-culture system that allows for the exchange of soluble factors between the two cell types without direct cell-cell contact (Additional file 1: Figure S1B). After 24 hours of macrophage priming, the priming medium was replaced with fresh RPMI complete medium and TFs were seeded in the inserts of the transwell plates (2.6 × 10^4^ cells/cm^2^). Control groups consisted of TFs alone, M0 macrophages alone, M1 macrophages alone, and M2 macrophages alone (Additional file 1: Figure S1B).

### The effect of ASCs on IL1-β-induced inflammation

To determine whether ASCs could modulate the effects of IL-1β on TFs, ASCs were co-cultured with TFs using transwell plates. Some studies indicate that MSCs may not constitutively express immunomodulatory factors; these studies suggest that IFNγ (typically produced by T lymphocytes) may act as an initiating stimulus for MSC immunosuppressive activity [23,33,34]. Thus, ASCs were pre-treated for 48 hours with 50 ng/ml IFNγ (R&D Systems) (activated) or left untreated (naïve). ASCs and TFs were co-cultured at a 1:1 cell density ratio (2.6 × 10^4^ cells/cm^2^) using six-well transwell plates (Costar, 0.4 μm pore size, Fisher Scientific, Pittsburgh, PA, USA). TFs were plated in the wells and ASCs were plated in the inserts. The cells were allowed 24 hours to attach before treatment with 10 ng/ml IL-1β (R&D Systems) on day 0. ASCs and TFs were co-cultured for one day. Experiments were run using 1% FBS as described previously. The experimental and control groups examined were: (1) TFs cultured alone (TT), (2) TFs treated with IL-1β (TT+), (3) TFs co-cultured with naïve ASCs (TA), (4) TFs co-cultured with naïve ASCs and treated with IL-1β (TA+), (5) TFs co-cultured with activated ASCs (TAa), and (6) TFs co-cultured with activated ASCs and treated with IL-1β (TAa+) (Additional file 1: Figure S1A). TFs were added to the inserts of groups 1 and 2 to maintain equal cell numbers in all groups (Additional file 1: Figure S1A).

### The effect of ASCs on macrophage-induced inflammation

To determine whether ASCs could modulate the effects of M0 or M1 macrophages on TFs, ASCs were incorporated into the system. After 24 hours of macrophage priming, the priming medium was replaced with fresh RPMI complete medium. ASCs were seeded directly on top of the pre-existing macrophages (1.2 × 10^4^ cells/cm^2^) and TFs were seeded in the inserts of the transwell plates (2.6 × 10^4^ cells/cm^2^) (Additional file 1: Figure S1B). Macrophages, ASCs, and TFs were tri-cultured for 24 hours (one day timepoint). To examine longer term effects of ASCs on the macrophages, a separate group was analyzed in which ASCs and macrophages were co-cultured for an additional four days before TFs were added to the culture for the final day (five day timepoint).

### Outcome measures

To determine the effects of inflammation on TFs and to examine the potential anti-inflammatory effects of ASCs, cell viability and changes in gene expression, protein expression, and surface marker expression were assessed.

#### Cell viability

To assess the viability of the TFs after IL-1β exposure, TFs were treated with 10 ng/ml of IL-1β or left untreated. One and three days after IL-1β treatment, the supernatant was removed and the cells were stained with a Live/Dead Viability/Cytotoxicity kit according to the manufacturer’s protocol (Invitrogen, Carlsbad, CA, USA). Cells were imaged using a fluorescent microscope and the percentage of live cells was calculated using ImageJ. Three regions of interest (4x objective) were imaged and their averages were calculated. The sampling method was validated prior to performing the analysis (there was less than 10% difference when comparing results from the entire well to results from three randomly selected regions of interest).

#### Gene expression of TFs and macrophages

To assess changes in gene expression of TFs under the various culture conditions, total RNA was isolated from the TFs (RNEasy Minikit, Qiagen, Valencia, CA, USA) on days 1 and 5. A total of 500 ng RNA was reverse transcribed to cDNA, using the Superscript VILO cDNA synthesis kit (Invitrogen Corporation). qRT-PCR was performed using SYBR Green chemistry on a StepOnePlus Real-Time PCR System (Applied Biosystems, Waltham, MA, USA) to measure the gene expression levels of factors related to inflammation (*IL-1β, TNFα, COX2*), matrix degradation (matrix metaloproteinases *MMP1a, 3, 13*), matrix production (collagens *COL1* and *COL3*, collagen fibrillogenesis regulators *BGN* and *DCN*), and TF differentiation (transcription factor *SCX*, TF marker *TNMD*). All primers were purchased from Qiagen (Additional file 1: Table S1). The data were analyzed using the delta delta Ct method, in which the data were normalized to the housekeeping gene (GAPDH) and then to the control group (that is, untreated TFs). To determine the effect of the different macrophage phenotypes on TF gene expression, delta Ct values were compared using an ANOVA, followed by a Fisher’s *post-hoc* test. To determine whether ASC co-culture had a significant effect on macrophage-induced gene expression by TFs, delta CT values were compared using a paired Student’s t-test.

To assess changes in gene expression of macrophages under the various culture conditions, total RNA was isolated from the TFs (RNEasy Minikit, Qiagen) on day 1. A total of 500 ng RNA was reverse transcribed to cDNA, using the Superscript VILO cDNA synthesis kit (Invitrogen). qRT-PCR was performed using TaqMan chemistry on a Fluidigm Biomark HD to measure the gene expression levels of macrophage-related factors (*IL-1β, IL-10, IL-12, IL-23, IL-1ra, TNF-α, Cxcl9, Ccl22, Arg1, MMP9,* and *TGFβ-1*; primers are detailed in Additional file 1: Table S2). The data were analyzed as described above for TFs.

#### Protein expression

To further examine the effects of inflammation and ASCs on TFs, the medium was collected on days 1 and 5 and the samples were stored at −80°C until analysis. The levels of IL-1β, TNFα, NO and PGE2 were assessed using commercially available kits, as described in the ‘Characterization of macrophage phenotype’ section above. Statistical differences were assessed using a two-way ANOVA (for macrophage type and presence of ASCs) followed by a Fisher’s *post-hoc* test.

#### Flow cytometry

The macrophage populations were further analyzed using flow cytometry to determine whether co-culture with ASCs led to a phenotypic switch in the macrophage population. Surface markers for all bone marrow-derived macrophages (CD11b+, F480+) and specifically for M2 macrophages (CD206+, CD301+) were assessed as described above. Statistical differences were assessed using a two-way ANOVA (for macrophage type and presence of ASCs) followed by a Fisher’s *post-hoc* test.

### Statistics

Flow cytometry data are expressed as geometric mean fluorescent intensity ± standard deviation. All other data are expressed as mean ± standard deviation, unless otherwise stated. Statistical differences were assessed using various ANOVAs (as described in the sections above), followed by a Fisher’s *post-hoc* test. Significance for all statistical analyses was set to *P* <0.05.

## Results

### Characterization of ASCs and induction of macrophage phenotypes

Flow cytometry results demonstrated that ASCs were positive for the MSC markers CD44 and CD29, and were negative for the hematopoietic markers CD14 and CD34 (Additional file 1: Figure S2). Recent data also verified the pluripotent capacity of these cells and their ability to regenerate and form colonies [32].

**Figure 1:**
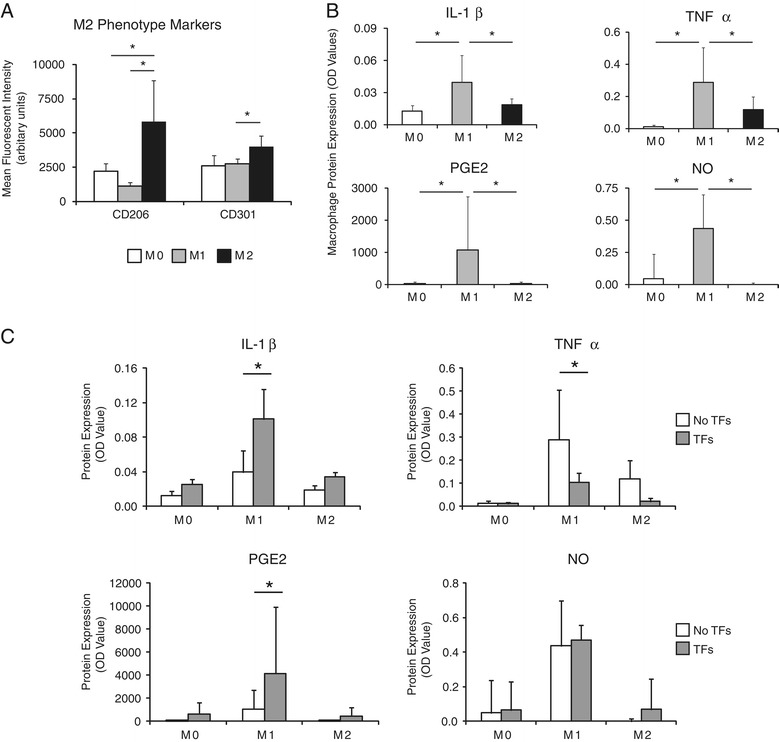
M0, M1, and M2 macrophage phenotypes were induced for the *in vitro* inflammation culture model. **(A)** Macrophage phenotypes were verified by examining expression of surface markers and expression of proteins one day after M1-priming, M2-priming, or no treatment (M0). Geometric mean fluorescent intensities of the M2 markers CD206 and CD301 were significantly increased in the M2 group compared to the M0 and M1 groups. **(B)** M1 macrophages expressed significantly higher levels of pro-inflammatory factors, IL-1β, TNFα, PGE2, and NO. There was a significant effect of macrophage type for all proteins. **(C)** Protein expression was determined by measuring the levels in the medium (that is, representing the cumulative expression from all cell types in a particular culture). M1 macrophages induced the secretion of inflammatory factors by TFs. Protein expression of IL-1β, TNFα, PGE2, and NO after one day of co-culture with M0, M1, or M2 macrophages in the presence and absence of TFs. There was a significant effect of TF for IL-1β, TNFα, and PGE2. Bars indicate significant differences (* *P* <0.05, N = 4 for flow cytometry, N = 5 for protein expression). Note that some data are repeated in (B) and (C) to highlight effects of macrophage type and TF, respectively. PGE2, prostaglandin E2; TF, tendon fibroblasts.

Flow cytometry revealed that all macrophages expressed high levels of CD11b and F480 (data not shown), and that macrophages treated with M2-priming medium also expressed high levels of CD206 and CD301 compared to M0 and M1 macrophages (Figure 1A). Geometric mean fluorescent intensities (MFI) of CD206 and CD301 were 2.6- and 1.5-fold greater, respectively, in the M2 macrophages compared to the M0 macrophages. Similarly, the MFIs of CD206 and CD301 were 5.1- and 1.5-fold greater, respectively, in the M2 macrophages compared to the M1 macrophages. To further evaluate macrophage phenotypes, protein expression was examined. M1 macrophages consistently secreted significantly greater amounts of inflammation-related proteins compared to M0 and M2 macrophages (Figure 1B). For example, M1 macrophages secreted approximately 4.0-, 29-, 29-, and 8.8- fold more IL-1β, TNFα, PGE2, and NO, respectively, than did M0 macrophages. Similarly, 2.0-, 2.4-, 26-, and 44- fold increases for these proteins, respectively, were seen relative to M2 macrophages (Figure 1B). When examining gene expression, M0 macrophages showed high expression of *TGFβ1* compared to M1 and M2 cells; M1 macrophages showed high expression of *IL23, Cxcl9*, and *Ccl22* compared to M0 and M1 cells; and M2 macrophages showed high expression of *IL1-ra*, *TNF-α, Arg1*, and *MMP9* compared to M0 and M1 cells (Additional file 1: Figure S3).

### Macrophages induced up-regulation of pro-inflammatory and matrix degradation factors by TFs

The effect of macrophages on TFs was determined by co-culturing TFs with the various macrophage phenotypes (Additional file 1: Figure S1). Gene expression analysis of TFs revealed that all three macrophage phenotypes induced up-regulation of pro-inflammatory factors (*TNFα, IL-1β*, and *COX2*) by TFs relative to untreated TFs (Table 1). Of the three phenotypes, however, the M1 macrophages had the largest effect on TFs compared to M0 and M2 macrophages. Co-culture with M1 macrophages led to a 2,800-fold increase in *IL-1β* by TFs, whereas co-culture with M0 and M2 macrophages led to only 21- and 86-fold increases, respectively.

**Table 1:** **Macrophages induced up-regulation of pro-inflammatory and matrix degradation factors and down-regulation of matrix production- and tendon differentiation-related factors by TFs**

**Day 1**	**M0**		**M1**		**M2**	
	**Mean**	***P*** **Value**	**Mean**	***P*** **Value**	**Mean**	***P*** **Value**
TNF	**20 ± 7.8**	**0.000**	**102 ± 86.3**	**0.001**	**49 ± 31.3**	**0.000**
IL-1β	**21 ± 12.3**	**0.001**	**2791 ± 1765.2**	**0.000**	**86 ± 117.9**	**0.002**
COX2	1.3 ± 0.8	0.942	**4.9 ± 4.0**	**0.032**	1.1 ± 0.6	0.915
MMP1a	**3.0 ± 1.3**	**0.008**	**150 ± 90.5**	**0.000**	**5.5 ± 5.3**	**0.011**
MMP3	6.3 ± 6.0	0.179	**111 ± 58.8**	**0.000**	**5.1 ± 3.8**	**0.054**
MMP13	1.8 ± 1.1	0.044	**46 ± 12.0**	**0.000**	**2.0 ± 0.8**	**0.027**
DCN	**−1.3 ± 0.1**	**0.019**	**4.3 ± 2.7**	**0.006**	−1.0 ± 0.3	0.689
BGN	**−1.4 ± 0.1**	**0.002**	**−1.7 ± 0.1**	**0.001**	**−1.4 ± 0.1**	**0.004**
COL1	**−1.7 ± 0.1**	**0.001**	**−3.4 ± 0.0**	**0.000**	**−1.9 ± 0.1**	**0.001**
COL3	**−1.3 ± 0.2**	**0.082**	**−2.8 ± 0.1**	**0.000**	**−1.6 ± 0.1**	**0.002**
SCX	1.2 ± 0.5	0.660	−1.3 ± 0.3	0.122	−1.2 ± 0.3	0.201
TNMD	1.0 ± 0.5	0.796	**−2.9 ± 0.2**	**0.003**	−1.4 ± 0.3	0.124

mRNA expression of TFs after one day of co-culture with macrophages (M0, M1, M2) are represented as fold changes compared to untreated TFs (mean ± SD). Of the three macrophage phenotypes, M1 macrophages had a significantly greater effect on TF gene expression than M0 or M2. There was a significant effect of macrophage type for all factors (indicated by bold font; N = 5). BGN, biglycan; COL, collagen; COX, cyclooxygenase; DCN, decorin; IL-1β, interleukin-1β; MMP, matrix metalloproteinase; SCX, scleraxis; TFs, tendon fibroblasts; TNF, tumor necrosis factor; TNMD, tenomodulin.

Exposure of TFs to macrophages also led to significant up-regulation of factors related to matrix degradation (that is, matrix-metalloproteinases (MMP)). TFs exposed to M1 macrophages expressed 150-, 110-, and 46-fold increases in *MMP-1a, 3, and 13*, respectively, compared to untreated TFs (Table 1). Similarly, M0 and M2 macrophages also induced significant up-regulation of MMP expression by the TFs; however, as with IL-1β, the up-regulation caused by M0 and M2 macrophages compared to control was significantly less than that caused by M1 macrophages (Table 1).

M1 macrophages also led to down-regulation of factors related to matrix production and TF differentiation by TFs. M1 macrophages induced down-regulation of collagens (*COL1, COL3*) and biglycan (*BGN*) and up-regulation of decorin (*DCN*) (Table 1). M1 macrophages caused a 3.4- and 2.8-fold decrease in *COL1* and *COL3*, respectively, by TFs and a 4.3-fold up-regulation of *DCN* (indicative of decreased collagen fibrillogenesis). M1 macrophages also had a significant effect on the tendon-specific gene, *TNMD* (2.9-fold decrease) (Table 1). M0 and M2 macrophages had little effect on the expression of matrix production- and TF differentiation-related genes by the TFs, except in the case of *COL1*. M0 and M2 macrophages caused 1.7- and 1.9-fold decreases in *COL1* expression by co-cultured TFs (Table 1).

When examining gene expression of macrophages, TFs caused an upregulation of: *IL-1β* in M0 and M2 cells, *IL-10* in M0 cells, *IL-12* in M0 cells, *IL-23* in M0 cells, *Ccl22* in M0 and M1 cells, and *Arg1* in M0 cells (Additional file 1: Figure S3). TFs caused a downregulation of: *IL-1ra* in M0 and M2 cells, *TNF-α* in M0 and M2 cells, *Cxcl* in M2 cells, *MMP9* in M0 cells, and *TGFβ-1* in M0 cells (Additional file 1: Figure S3).

At the protein level, co-cultures of M1 macrophages with TFs led to higher levels of IL-1β and PGE2 protein secretion compared to M1 macrophages alone (2.9- and 1.8-fold, respectively) (Figure 1C). Since TFs do not secrete IL-1β and PGE2 under normal culture conditions, this result indicates that the presence of M1 macrophages induced TFs to secrete some pro-inflammatory factors or that TFs secreted factors that increased cytokine production by macrophages. In contrast, co-cultures of M1 macrophages with TFs led to lower levels of the cytokine TNFα and had no effect on NO production (Figure 1C).

### IL-1β induced expression of inflammation and matrix degradation genes by TFs

**Figure 2:**
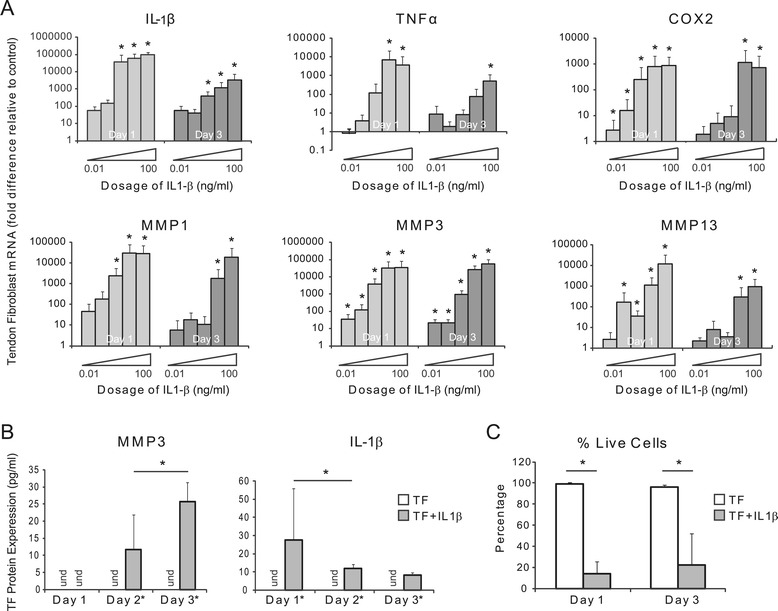
IL-1β induced the production of inflammation-related factors by TFs. **(A)** IL-1β-induced up-regulation of inflammation-related genes by TFs occurs in a dose- and time-dependent manner. Gene expression for each dosage is normalized to control (0 ng/ml) (* *P* <0.05, multi-factor ANOVA, N = 4, except for IL-1β on day 1, N = 3). **(B)** IL-1β (10 ng/ml) induced MMP3 and IL-1β protein expression by TF (und = undetected, * *P* <0.05, ANOVA, significant effect of IL-1β treatment and time, N = 4). **(C)** IL-1β (10 ng/ml) led to decreased TF viability (* *P* <0.05, two-way ANOVA, significant effect of IL-1β treatment, N = 3). ANOVA, analysis of variance; MMP3, matrix metalloproteinase 3; TF, tendon fibroblasts.

In order to examine the isolated effect of a single inflammatory cytokine, known to play a role in tendon-related inflammation, TFs were exposed to IL-1β *in vitro*. TFs responded to IL-1β by up-regulating inflammatory (*IL-1β, TNFα,* and *COX2*) and matrix degradation (*MMP1, MMP3*, and *MMP13*) genes in a dose dependent manner (Figure 2A) at one and three days of culture. IL-1β also led to a decrease in the expression of extracellular matrix genes by TFs (Additional file 1: Figure S4). At the protein level, TFs cultured in the absence of IL-1β did not secrete detectable levels of IL-1β or MMP3 (Figure 2B). However, 10 ng/ml of IL-1β induced the production of MMP-3 by TFs in a time-dependent manner (Figure 2B). Similar trends were observed for IL-1β. There were no time-dependent increases in IL-1β levels, however, suggesting that increased IL-1β on the first day was due to the exogenous addition of IL-1β rather than production of the cytokine by TFs. TNFα remained undetectable in cultures with and without IL-1β (data not shown). Cell viability was significantly reduced due to IL-1β (Figure 1C). On days one and three, respectively, 96% and 99% percent of TFs remained viable in the control cultures whereas only 14% and 22%, respectively, were viable in the IL-1β-treated cultures.

### ASCs suppressed the negative effects of macrophages on TFs but did not directly modulate the response of TFs to IL-1β

**Figure 3:**
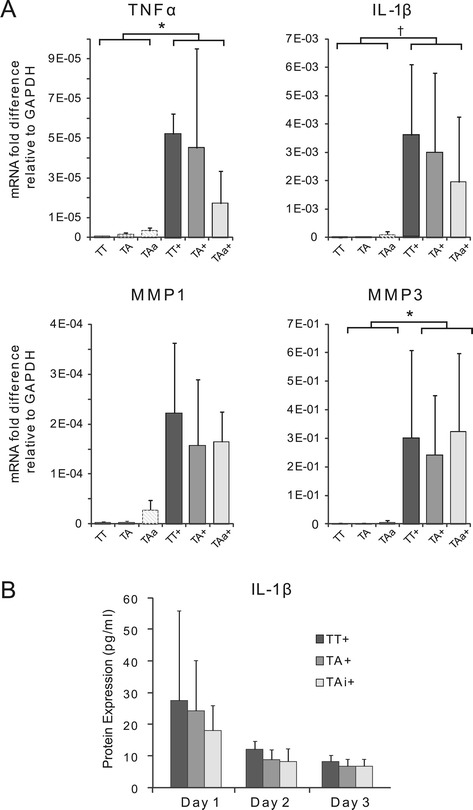
ASCs did not suppress the negative effects of IL-1β on TFs. **(A)** ASCs failed to suppress the IL-1β-induced effects on TF gene expression. TFs were cultured alone (TT), co-cultured with naïve ASCs (TA), or co-cultured with activated ASCs (TAa; activated with IFNγ), with or without IL-1β for one day (inclusion of IL-1β indicated with ‘+’). Data were normalized to the housekeeping gene GAPDH (* *P* <0.05, † *P* <0.10, based on a multi-factor ANOVA with Fisher’s *post-hoc* tests there was a significant effect of IL-1β but no effect of ASCs, N = 4). **(B)** ASCs failed to reduce IL-1β protein levels in the medium after one, two, and three days of co-culture (based on a multi-factor ANOVA and Fisher’s *post hoc* tests there was a significant effect of time but no effect of ASCs, N = 4). (TT: TFs cultured alone, TT+: TFs treated with IL-1β, TA: TFs co-cultured with naïve ASCs, TA+: TFs co-cultured with naïve ASCs and treated with IL-1β, TAa: TFs co-cultured with activated ASCs, TAa+: TFs co-cultured with activated ASCs and treated with IL-1β). ANOVA, analysis of variance; ASCs, adipose-derived mesenchymal stromal cells; TFs, tendon fibroblasts.

There was a dramatic upregulation of inflammation-related genes by TFs when they were exposed to IL-1β. TFs co-cultured with naïve ASCs did not change their gene expression or protein production levels (Figure 3, Additional file 1: Figure S5). Co-culture with activated ASCs led to a 3.1-, 1.8-, and 1.4-fold down-regulation of *TNFα, IL-1β*, and *MMP1* mRNA, respectively, on day 1; however, these reductions were not statistically significant. Thus, neither naïve ASCs nor activated ASCs modulated TF responses to IL-1β (Figure 3). Furthermore, there were no apparent effects of ASCs on TF gene expression under standard culture conditions without IL-1β (Figure 3, Additional file 1: Figure S5).

**Figure 4:**
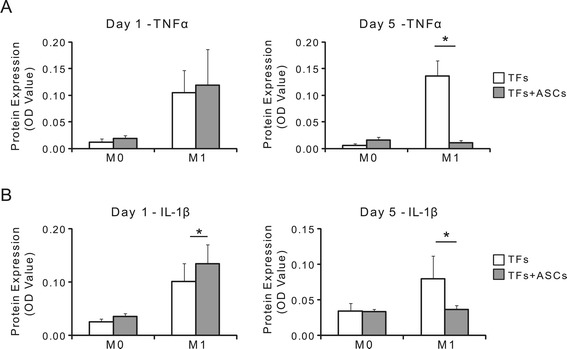
Protein expression of inflammatory factors **(A)** TNFα and **(B)** IL-1β was determined after one or five days for TFs cultured with macrophages with and without ASCs. ASCs suppressed the effects of M1 macrophages on the secretion of pro-inflammatory factors after five days of co-culture. There was a significant effect of macrophage type for both factors at both timepoints, and a significant effect of ASCs on day 5 for both factors (* *P* <0.05; N = 5 to 6 for day 1, N = 4 for day 5). ASCs, adipose-derived mesenchymal stromal cells; TFs, tendon fibroblasts.

The response of TFs to M1 macrophage, however, was modulated by ASCs. The addition of ASCs to the M1 macrophage-TF co-culture significantly altered TF gene expression compared to M1 macrophage-TF co-culture (Table 2, Additional file 1: Figure S6). *TNFα* gene expression by TFs was down-regulated 1.7- and 1.9-fold after one and five days of ASC co-culture, respectively. While changes failed to reach statistical significance at the gene expression level for the five day timepoint, significant reductions were observed at the protein level (Figure 4). *IL-1β* gene and protein expression were not affected after a single day of co-culture (Table 2, Additional file 1: Figure S6). Five days of ASC co-culture with M1 macrophages, however, led to a 1.8-fold down-regulation of the *IL-1β* gene by TFs, although this change was not statistically significant. A corresponding significant two-fold reduction in IL-1β was seen at the protein level (Figure 4). *COX2* was unaffected after one day of co-culture, but a significant 10-fold down-regulation was observed after five days of co-culture (Table 2, Additional file 1: Figures S6). No significant effect was seen at the protein level, however (data not shown).

**Table 2:** **ASCs suppressed the effects of M1 and M0 macrophages on TF secretion of pro-inflammatory factors**

**Day 1**	**M0 versus M0 + ASCs**		**M1 versus M1 + ASCs**	
	**Mean**	***P*** **Value**	**Mean**	***P*** **Value**
TNF	1.8 ± 1.6	0.620	−1.7 ± 0.3	0.060
IL-1b	3.4 ± 3.4	0.340	1.0 ± 0.6	0.510
COX2	−1.2 ± 0.2	0.230	−1.1 ± 0.3	0.440
MMP1a	−1.9 ± 0.2	0.020	−1.9 ± 0.3	0.060
MMP3	−1.9 ± 0.2	0.010	−1.1 ± 0.2	0.350
MMP13	−1.3 ± 0.6	0.220	−1.5 ± 0.3	0.150
COL1	1.3 ± 0.3	0.030	1.1 ± 0.3	0.660
COL3	1.3 ± 0.5	0.280	−1.1 ± 0.2	0.440
BGN	1.1 ± 0.1	0.250	−1.1 ± 0.3	0.520
DCN	1.2 ± 0.4	0.440	−1.2 ± 0.3	0.240
SCX	−1.8 ± 0.1	<0.001	−1.1 ± 0.1	0.100
TNMD	−1.5 ± 0.2	0.030	1.0 ± 0.1	0.580
**Day 5**	**M0 versus M0 + ASCs**		**M1 versus M1 + ASCs**	
	**Mean**	***P*** **Value**	**Mean**	***P*** **Value**
TNF	2.7 ± 1.9	0.160	−1.9 ± 0.3	0.140
IL-1b	3.7 ± 3.3	0.180	−1.8 ± 0.6	0.280
COX2	−1.0 ± 0.1	0.760	−10 ± 0.0	<0.001
MMP1a	−1.5 ± 0.3	0.240	−7.7 ± 0.1	0.060
MMP3	1.5 ± 0.2	0.050	−4.6 ± 0.2	0.050
MMP13	1.9 ± 0.4	0.040	5.0 ± 6.6	0.490
COL1	1.0 ± 0.1	0.940	1.8 ± 0.1	<0.001
COL3	1.4 ± 0.3	0.290	2.0 ± 0.8	0.140
BGN	1.3 ± 0.3	0.390	−1.2 ± 0.2	0.300
DCN	1.2 ± 0.1	0.280	1.0 ± 1.1	0.770
SCX	1.2 ± 0.1	0.150	−1.8 ± 0.1	0.030
TNMD	1.4 ± 0.1	0.100	1.2 ± 0.3	0.430

ASCs had the greatest effect on M1 macrophages on day 5. Gene expression of inflammation-, degradation-, matrix production-, and tendon differentiation-related factors after one day (top) and five days (bottom) of co-culture with macrophages (M0, M1) or tri-culture with macrophages and ASCs (M0 + ASC, M1 + ASC). Data from each group were normalized first to untreated TFs and then to their paired control (that is, (M0 + ASC)/M0, (M1 + ASC)/M1). Data are presented as mean ± SD; N = 5 for day 1, N = 2 to 3 for day 5. This data is presented graphically in the supplemental document, Additional file 1: Figure S6. ASCs, adipose-derived mesenchymal stromal cells; biglycan; COL, collagen; COX, cyclooxygenase; DCN, decorin; IL-1β, interleukin-1β; MMP, matrix metalloproteinase; SCX, scleraxis; TFs, tendon fibroblasts; TNF, tumor necrosis factor; TNMD, tenomodulin.

MMP gene expression by TFs in the presence of M1 macrophages was also suppressed by addition of ASCs. *MMP1a* expression by TFs was down-regulated 1.9- and 7.7-fold after one and five days of culture with ASC, respectively (Table 2, Additional file 1: Figure S6). *MMP3* expression was unaffected after a single day of culture with ASCs; however, five days of culture with ASCs led to a significant 4.6-fold down-regulation. No effects on *MMP13* expression were observed at either timepoint with the addition of ASCs (Table 2, Additional file 1: Figure S6).

Extracellular matrix (ECM)-related gene expression for *COL1* and *COL3* by TFs in the presence of M1 macrophages was unaffected after one day of culture with ASCs, but was up-regulated 1.8- and 2.0-fold, respectively, towards baseline after five days of culture with ASCs (Table 2, Additional file 1: Figure S6). However, this change failed to reach statistical significance for *COL3*. No effects on TF differentiation genes (that is, *SCX, TNMD*) was seen at the one day timepoint, but *SCX* was down-regulated 1.8-fold on day 5 with the addition of ASCs (Table 2, Additional file 1: Figure S6).

ASCs blunted some of the effects of macrophages on TF gene expression. Addition of ASCs to the TF/macrophage co-culture led to decreased expression of matrix degradation genes (*MMP1a, MMP3*) and increased expression of matrix- and tendon differentiation-related genes (that is, *COL1, SCX, TNMD*) on day 1, but failed to suppress the inflammation-related genes (that is, *TNFα, IL-1β*) (Table 2, Additional file 1: Figure S6). Longer duration of ASC co-culture (that is, five days) was unsuccessful in further suppressing these effects (Table 2, Additional file 1: Figure S6). Similarly, no differences were observed at the protein level (Figure 4).

### ASCs promoted an M2 macrophage phenotype

**Figure 5:**
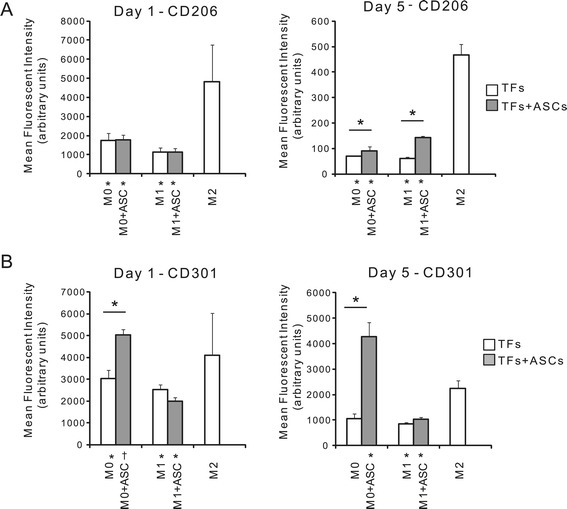
The addition of ASCs to macrophage-TF cultures led to a shift in macrophage phenotype toward M2. Mean fluorescent intensity of M2 macrophage-specific surface markers **(A)** CD206 and **(B)** CD301 is shown after one or five days of culture. Macrophages (M0, M1, or M2) were either co-cultured with TFs for one day or tri-cultured with TFs and ASCs for one or five days (note that TFs were only added for the last 24 hours). There was a significant effect of macrophage type for CD206 and CD301, a significant effect of ASCs for CD301 on day 1 and day 5, and a significant effect of ASCs for CD206 on day 5 (* *P* <0.05, † *P* <0.10; */† by the x-axis labels signifies a significant difference compared to M2 macrophages cultured alone; N = 5). ASCs, adipose-derived mesenchymal stromal cells; TFs, tendon fibroblasts.

To examine a potential mechanism by which ASCs co-culture suppressed the negative effects of M1 and M0 macrophages on TFs, the phenotype of the co-cultured macrophages was determined using flow cytometry. CD11b and F480 are expressed by all bone marrow-derived macrophages [35]. In contrast, CD206 and CD301 are cell surface markers specific to M2 macrophages [36]. The addition of ASCs to M0 macrophage-TF cultures for a single day led to a two-fold increase in CD301 expression, surpassing the levels of M2 macrophage controls; however, no shift in CD206 expression was observed (Figure 5). No apparent phenotypic changes were evident with addition of ASCs after a single day (Figure 5). Increasing the ASC co-culture period to five days, however, led to significant phenotypic changes in both M0 and M1 macrophages. The addition of ASCs to M0 macrophage-TF cultures for five days led to significantly greater levels of both CD206 and CD301 compared to M0 macrophage controls (1.3- and 4.1-fold, respectively) (Figure 5). Similarly, the addition of ASCs to M1 macrophage-TF cultures for five days led to a significant 2-fold increase in the expression of CD206 compared to M1 macrophage controls (Figure 5).

## Discussion

Tendon injuries induce a local inflammatory response characterized by infiltration of macrophages, release of pro-inflammatory cytokines, and increased matrix degradation [17,18,37]. Animal models have suggested that M1 macrophages contribute to the poor healing response of tendon to bone and that suppression of M1 macrophages can be advantageous for tendon healing [11,13,14]. *In vitro* evidence also supported the idea that poor healing was due in part to the harmful effects of pro-inflammatory factors such as IL-1β and TNFα secreted by M1 macrophages [19-22]. In support of these prior studies, findings from the current *in vitro* study demonstrate the detrimental effects of IL-1β and M1 macrophages on TFs. Furthermore, we show that ASCs can modulate the negative effects of M1 macrophages on TFs, revealing a new potential therapeutic option for the treatment of tendon injuries.

It is widely accepted that MSCs have anti-inflammatory and immunosuppressive capabilities [23,24,29,31,33,34,38-42]. The mechanism(s) by which MSCs regulate the inflammatory environment, however, remain unclear. Possibilities include: (1) MSC secretion of factors that modulate macrophage activity (for example, promoting changes in macrophage differentiation or cytokine expression patterns), (2) MSC secretion of factors that modulate TF activity (for example, leading to reduced sensitivity to cytokines such as IL-1β), or (3) MSC inactivation of circulating pro-inflammatory factors directly (for example, by releasing factors that degrade or sequester circulating pro-inflammatory cytokines). The results of the current study support the premise that MSCs can modulate macrophage activity, specifically prompting a phenotypic switch towards the M2 phenotype. In contrast, MSCs did not have a demonstrable effect on IL-1β-induced inflammation, implying that they neither modulate TF responses to inflammation nor do they inactivate circulating IL-1β (albeit, the current study examined TF-ASCs cultured in a transwell system, and did not examine TF-ASCs cultured in direct contact). Other reports have also suggested that MSCs modulate activity of inflammatory cells (and their production of inflammatory factors) via MSC-secreted cytokines [29,31]. Bone marrow-derived MSCs have been shown to drive monocyte differentiation toward an anti-inflammatory M2 phenotype instead of the classical pro-inflammatory M1 phenotype [29-31,43-45]. *In vitro,* non-polarized macrophages (M0s) co-cultured with MSCs consistently expressed high levels of CD206 (an M2 marker) and IL-10 (an anti-inflammatory factor) and low levels of IL-12 and TNFα (pro-inflammatory factors), indicating an MSC-mediated shift in macrophage phenotype from M0 to M2 [29]. This phenomenon has also been observed *in vivo* in a spinal cord injury model [30].

The ability of ASCs to prompt M0 macrophages toward an M2 phenotype is particularly attractive from the therapeutic perspective, as the vast majority of macrophages at the site of wound repair are recruited from the bone-marrow as undifferentiated monocytes. Thus, early treatment with ASCs could potentially promote differentiation of the infiltrating monocytes towards the anti-inflammatory M2 macrophage lineage as opposed to the pro-inflammatory M1 macrophage lineage. While ASCs appeared to have a more limited ability to alter M1 macrophage phenotype, the gene expression data in the current study suggests that ASCs were capable of suppressing the negative effects caused by M1 macrophages on TFs. Thus, even if treatment was delayed until after monocytes differentiated into M1 macrophages, ASCs may still be effective in modulating the inflammatory environment and making it more conducive to regenerative tendon healing.

The results of the current study are in agreement with those of other investigators. Kim *et al*. revealed an increase in CD206 expression by non-polarized macrophages (that is, M0 macrophages) after four to five days of direct or indirect co-culture with bone marrow-derived MSCs [29]. The authors also identified altered intracellular cytokine staining after MSC co-culture via flow cytometry [29]. Similarly, in the current study CD206 expression was increased in M0 macrophages after five days of direct ASC co-culture and a significant decrease in TNFα secreted into the medium was observed after five days of MSC co-culture with M0 macrophages. We also examined a second M2-specific surface marker, CD301, which was not investigated by Kim *et al*. Expression of CD301 was increased in M0 macrophages after five days of ASC co-culture to levels that surpassed those of M2 macrophage controls. Finally, although ASC co-culture successfully suppressed some of the negative effects of M1 macrophages on TF gene expression in the current study, ASC co-culture was not as successful in altering the phenotype of M1 macrophages. In other words, once macrophages were fully differentiated into an M1 phenotype, they remained in that state.

In the current study, exposure of TFs to IL-1β was detrimental to TFs. IL-1β exposure resulted in a decline in TF viability, up-regulation of genes related to inflammation and matrix degradation, and down-regulation of factors related to tendon ECM and differentiation. These results are consistent with those of previous studies reporting harmful effects of IL-1β and TNFα on TFs when the cytokine was added directly to the culture media [19-22]. This approach allowed for a well-controlled environment where the levels of inflammatory cytokines could be tightly titrated and the hypothesis, related to direct regulation of TFs and/or circulating cytokines by ASCs, tested. However, it did not reproduce the complex environment of a healing tendon, which includes multiple cell types. As macrophages are the primary source of IL-1β and TNFα and this cell type has been identified in various tendinopathy and tendon healing models [11,13,14,18,46-48], we developed a more biologically relevant co-culture *in vitro* model for the current study. The data revealed that, among the three macrophage phenotypes examined, M1 macrophages produced the most harmful effects on TFs. These findings are consistent with increased production of cytokines such as IL-1β and with the results from the IL-1β-induced inflammation cultures. Importantly, the use of transwell plates for the exchange of soluble factors between the various cell types, allowed for the specific evaluation of TF responses. Results are also consistent with Al-Sadi *et al*., who showed in a leukocyte/TF co-culture model that leukocytes upregulated inflammatory cytokine and MMP gene expression in TFs [22].

Gene expression was only examined in TFs and macrophages. In some experiments, macrophages and ASCs were co-cultured in contact, so their differential gene expression could not be separated. Similarly, protein expression was measured from the medium. As soluble factors released by any of the three cell types would contribute to this measure, the source of protein expression could not be isolated. Furthermore, TFs prompted changes in the expression patterns of various macrophage phenotypic markers (Additional file 1: Figure S3). For example, co-culture with TFs led to increased expression of IL-1β by M0 and M2 macrophages. This indicates that there is significant crosstalk between the three cell types studied here, each one likely regulating the expression of the other two. Further study is needed to determine the separate effects of TFs on macrophages, of TFs on ASCs, of ASCs on macrophages, and of macrophages on ASCs. Macrophages, for example, have been shown to modulate the viability and growth of MSCs, with M2 macrophages supporting growth and M1 macrophages inhibiting growth [49].

There are limitations to our study. First, it is unclear whether or not the increases in IL-1β and PGE2 protein in the co-culture models were due to increased synthesis and secretion of those factors by the TFs or the presence of the TFs caused increased synthesis and secretion of those factors by the M1 macrophages. Based on the observation that the expression levels for the corresponding genes (*IL-1β* and *COX2*) were similarly up-regulated by TFs, it is likely that the source of the additional IL-1β and PGE2 protein was at least, in part, produced by TFs. Second, we did not perform assays for MMP protein activity (that is, zymography). Thus, it remains unclear whether or not the up-regulation of MMP genes resulted in increased MMP activity. Third, the data from the ASC co-cultured groups were obtained on day 5, whereas the data from the control groups (M0, M1, and M2) were obtained on day 1. Thus, we are unable to say with certainty that the enhanced effects that were noted with the increased culture interval were due entirely to increased time for ASCs to induce phenotypic changes in the co-cultured macrophages, as there was likely a decrease in macrophage numbers at the five day timepoint (as observed by decreased adhered cells and debris floating in the medium). To better control for this possibility, the control groups were repeated at longer timepoints. The macrophages did not survive for five days in mono-culture, however. While this timing issue is a limitation of this study, a number of observations support the concept that longer periods of ASC exposure can lead to M2 phenotypes and, subsequently, to lower levels of inflammation- and matrix remodeling-related genes and to higher levels of tendon extracellular matrix production. Specifically, (1) CD206 and CD301 levels increased over time, (2) TNFα and IL1-β protein levels were markedly reduced when comparing TF + M1 to TF + M1 + ASCs, (3) mRNA expression for genes such as *MMP1a* was down-regulated by M0 and M1 cells when cultured with ASCs, including at day 1, and (4) M2 phenotype markers were increased when M0 cells were cultured with ASCs, including at day 1. We did not systematically test the effect of culture in the top versus bottom chamber or direct versus indirect cell contact. In pilot experiments for this study, and in the TF/TF control group (Additional file 1: Figure S1, A, top row), there were no apparent differences between culturing TFs in the top versus bottom chamber. Macrophages were always cultured in the bottom chamber (Additional file 1: Figure S1, B), and ASCs were cultured in the top chambers for the exogenous IL-1β experiments (Additional file 1: Figure S1, A) and in the bottom chamber (in contact with macrophages) for macrophage experiments (Additional file 1: Figure S1, B). Testing all possible permutations for variations in chamber location, cell-cell contact, and other culture details would quickly result in an unwieldy study design. We therefore chose the study design in the current paper with these limitations in mind, and will pursue effects of uncontrolled variables, such as cell-cell contact, in future studies.

## Conclusions

Overall, this study supports the conclusion that M1 macrophages and the pro-inflammatory cytokine IL-1β, in particular, are detrimental to TFs, with negative implications for tendon healing. Moreover, there is strong evidence to support the notion that ASCs can prompt pro-inflammatory M1 macrophages towards anti-inflammatory M2 macrophages. Furthermore, if ASC treatment is performed prior to monocyte differentiation into M1 macrophages, ASCs may be able to further modulate the inflammatory environment by pushing the undifferentiated (M0) macrophages towards an M2 phenotype. Our results suggest that ASCs may be able to suppress the negative effects of M1 macrophages, even if the treatment is delayed until after monocytes have differentiated into M1 macrophages. As with any *in vitro* study, the results presented here must be validated using *in vivo* models. Future studies will determine if ASCs delivered at the time of tendon repair can enhance tendon healing via modulation of the early inflammatory phase of healing.

## Additional file

Additional file 1:
**Supplemental tables and figures for the manuscript.** **Table S1:** Primers used for TF qRT-PCR. **Table S2:** Primers used for macrophage qRT-PCR. **Figure S1:** Schematics of *in viro* experimental design. **Figure S2:** ASCs characterization using flow cytometry. **Figure S3:** Macrophage gene expression. **Figure S4:** Changes in TF gene expression due to IL-1β. **Figure S5:** ASCs failed to suppress the IL-1β-induced effects on TF gene expression. **Figure S6:** Changes in gene expression for TFs after culture with macrophages with and without ASCs.

## Abbreviations

ANOVA: analysis of variance
Arg1: arginase 1
ASC: adipose derived stromal cell
BGN: biglycan
CCL22: C-C motif chemokine 22
COL: collagen
COX2: cyclooxygenase 2
Cxcl9: chemokine (C-X-C motif) ligand 9
DCN: decorin
ELISA: enzyme-linked immunosorbent assay
FBS: fetal bovine serum
GAPDH: glyceraldehyde 3-phosphate dehydrogenase
IFN: interferon
IL: interleukin
IL-1β: interleukin 1 beta
MFI: mean fluorescent intensity
MMP: matrix metalloproteinase
MSC: mesenchymal stromal cells
NO: nitric oxide
PGE2: prostaglandin E2
RPMI: Roswell Park Memorial Institute
SCX: scleraxis
TF: tendon fibroblast
TGFβ-1: transforming growth factor β 1
TNFα: tumor necrosis factor alpha
TNMD: tenomodulin
