# Supplementary material for: Adipose-derived mesenchymal stromal cells modulate tendon fibroblast responses to macrophage-induced inflammation in vitro
Source: Stem Cell Res Ther. 2015 Apr 16;6(1):74. doi: 10.1186/s13287-015-0059-4 (PMC4416344; doi:10.1186/s13287-015-0059-4)
Supplement: Additional file 1: — Supplemental tables and figures for the manuscript. Table S1: Primers used for TF qRT-PCR. Table S2: Primers used for macrophage qRT-PCR. Figure S1: Schematics of in viro experimental design. Figure S2: ASCs characterization using flow cytometry. Figure S3: Macrophage gene expression. Figure S4: Changes in TF gene expression due to IL-1β. Figure S5: ASCs failed to suppress the IL-1β-induced effects on TF gene expression. Figure S6: Changes in gene expression for TFs after culture with macrophages with and without ASCs. [file 13287_2015_59_MOESM1_ESM.pdf]

Adipose-derived mesenchymal stromal cells modulate tendon fibroblast responses to macrophage-induced inflammation

- Supplemental Document -

Manning, CN[a], Martel, C[b], Sakiyama-Elbert, S[c], Silva, MJ[a], Shivam Shah[a], Gelberman, RH[a], Thomopoulos, S[a]*

[a]Department of Orthopaedic Surgery, Washington University, 425 S Euclid, Box 8233, St Louis, MO 63110, USA

[b]Department of Pathology and Immunology, Washington University, 425 S Euclid, Box 8233, St Louis, MO 63110, USA

[c]Department of Biomedical Engineering, Washington University, One Brookings Drive, Campus Box 1097, Washington University, St Louis, MO, USA

*Corresponding Author:
Stavros Thomopoulos, Ph.D.
Washington University
660 South Euclid, Campus Box 8233
St Louis, MO 63110, USA
Phone: +1-314-362-8605
Fax: +1-314-362-0334, Email: ThomopoulosS@wudosis.wustl.edu

Table S1: Primers used for TF qRT-PCR.

Gene	QuantiTect Primer
IL-1β	QT01048355
TNFα	QT00104006
Cox2	QT00165347
MMP1a	QT00138894
MMP3	QT00107751
MMP13	QT00111104
Col1	QT0016220
Col3	QT02331301
Bgn	QT00108682
Dcn	QT00131068
Scx	QT00166271
Tnmd	QT00126427
GAPDH	QT01658692

Table S2: Primers used for macrophage qRT-PCR.

Gene	TaqMan Primer
IL-1β	Mm00434228_m1
TNFα	Mm00443258_m1
IL-1Ra	Mm00446186_m1
IL-10	Mm00439614_m1
IL-12	Mm00434174_m1
IL-23	Mm01160011_g1
Cxcl9	Mm00434946_m1
CCL22	Mm00436439_m1
Arg1	Mm00475988_m1
MMP9	Mm00442991_m1
TGFβ-1	Mm01178820_m1
GAPDH	Mm99999915_g1

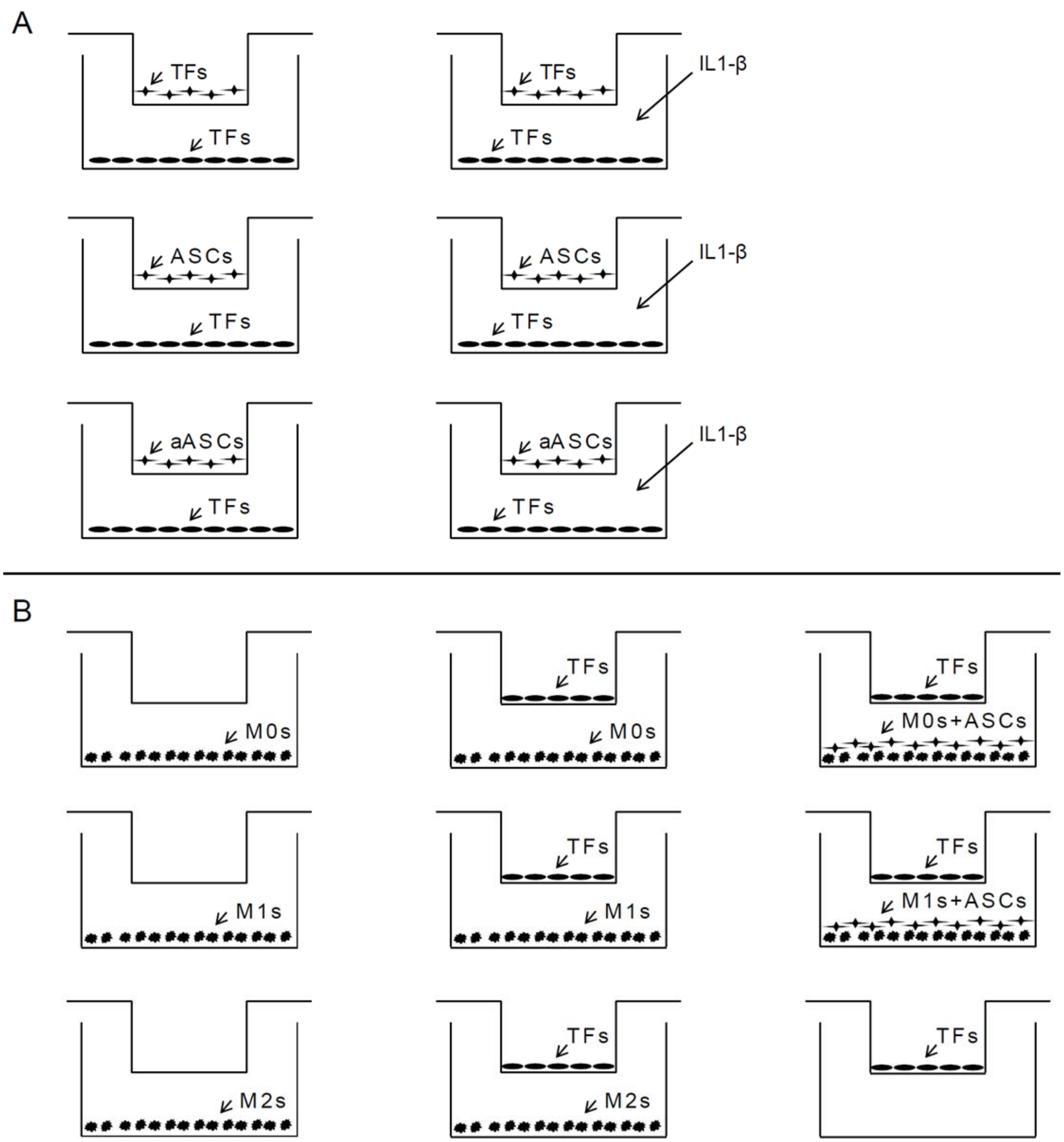

Figure S1: Schematics of *in viro* experimental design. (A) Schematic of experimental design for the IL-1β-induced inflammation model. TFs were cultured alone or co-cultured with either naïve ASCs (ASCs) or activated ASCs (aASCs) in the presence of IL-1β. Controls consisted of the same experimental groups, but in the absence of IL-1β. Note: TFs were cultured in the inserts of the transwell plates for some groups to keep the total number of cells uniform between all groups. **(B)** Schematic of experimental setup for the macrophage-induced inflammation model. M0, M1, and M2 macrophages were cultured alone to characterize macrophage phenotype. TFs were co-cultured with M0, M1, or M2 macrophages to determine the effects of macrophages on TFs. TFs were cultured with ASCs and either M0 or M1 macrophages to determine if ASCs can protect TFs from the effects of macrophages. TFs also cultured alone to serve as controls.

A

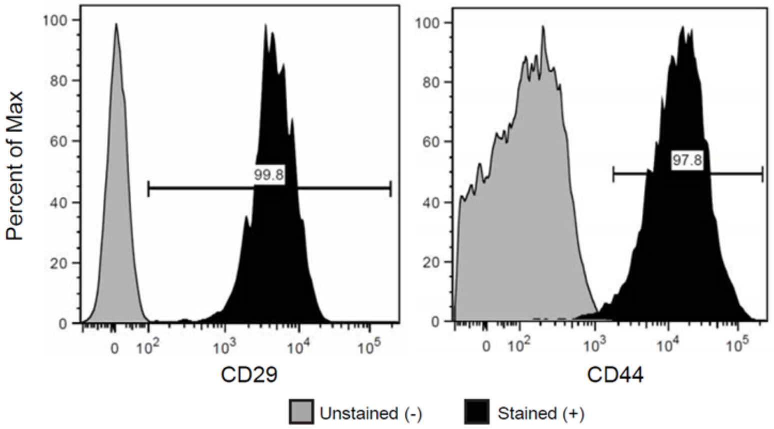

B

Percent Positive		
**Antibody**	**Mean ± SD**	**P Value**
CD44	96.4 ± 3.4	< 0.001
CD29	100 ± 0.05	< 0.001
CD14	0.46 ± 0.68	0.269
CD34	0.00 ± 0	0.218

Figure S2: ASCs characterization using flow cytometry. ASCs were labeled with antibodies against mesenchymal stem cell markers (CD29, CD44) and hematopoietic stem cell markers (CD14, CD34). The percentage of cells staining positive for the various markers was determined by comparing the expression of the stained ASCs to unstained cells. **(A)** Results are shown for representative samples. **(B)** The data are presented as mean percent positive ± SD (N=4).

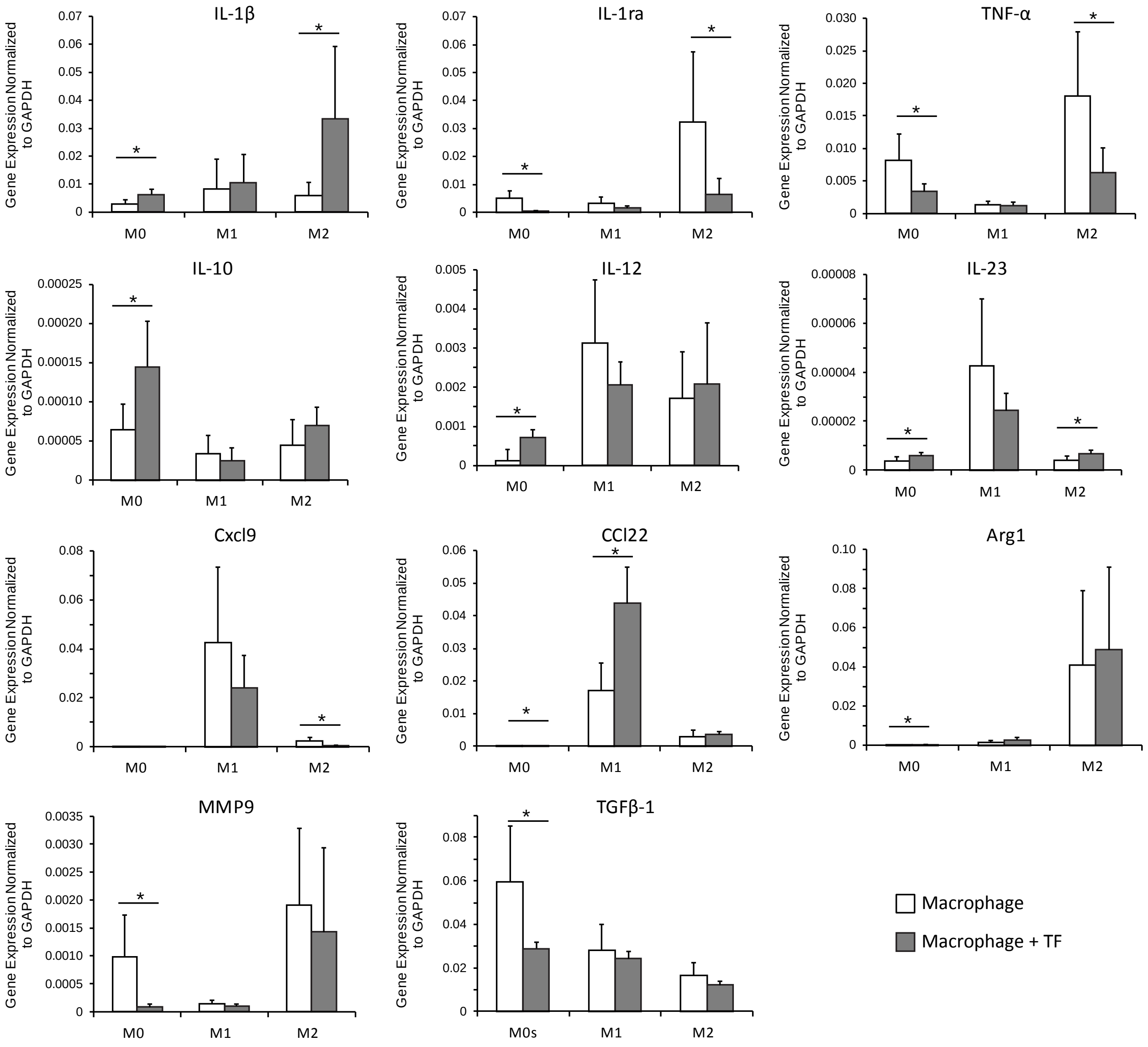

Figure S3: Macrophage gene expression. Changes in gene expression for macrophages was examined after culture alone (white bars) or co-culture with TFs (gray bars) for 1 day. There were significant effects of macrophage phenotype and presence of TFs on macrophage gene expression. Gene expression was normalized to GAPDH (* $p < 0.05$, N=10 for macrophage alone and N=6 for macrophage/TF co-culture).

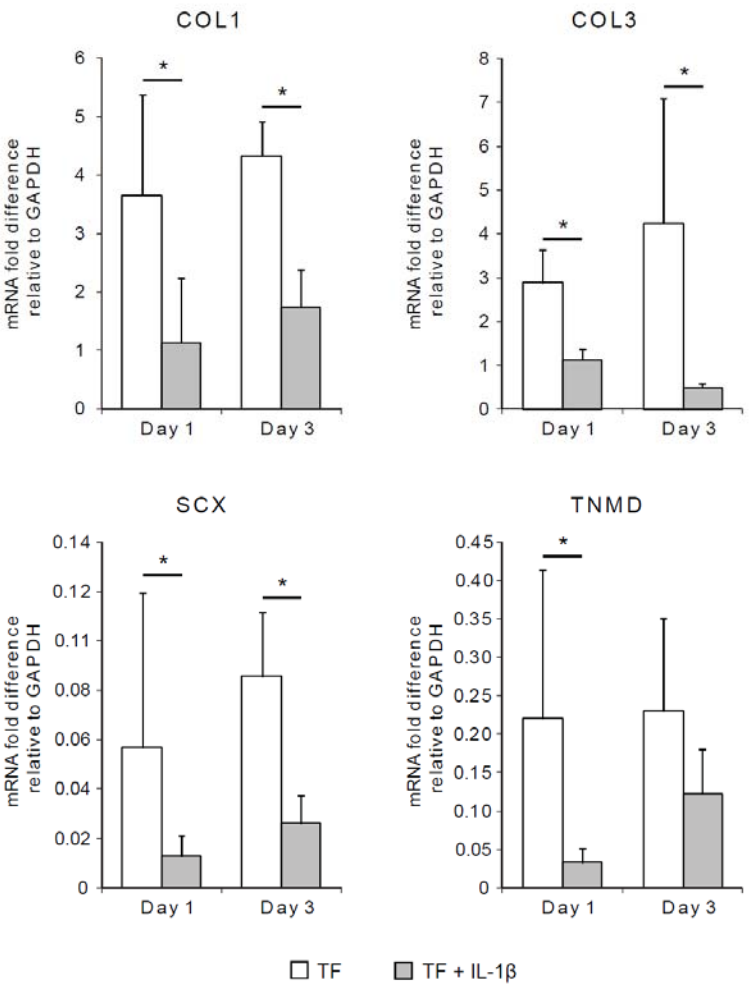

Figure S4: Changes in TF gene expression due to IL-1β. IL-1β induced down-regulation of tendon ECM- and differentiation-related genes by TFs. Results are shown for tendon ECM (COL1, COL3) and differentiation (SCX, TNMD) genes for TFs and in TFs treated with 10 ng/ml of IL-1β for 1 or 3 days (* $p < 0.05$, two-way ANOVA, N=4).

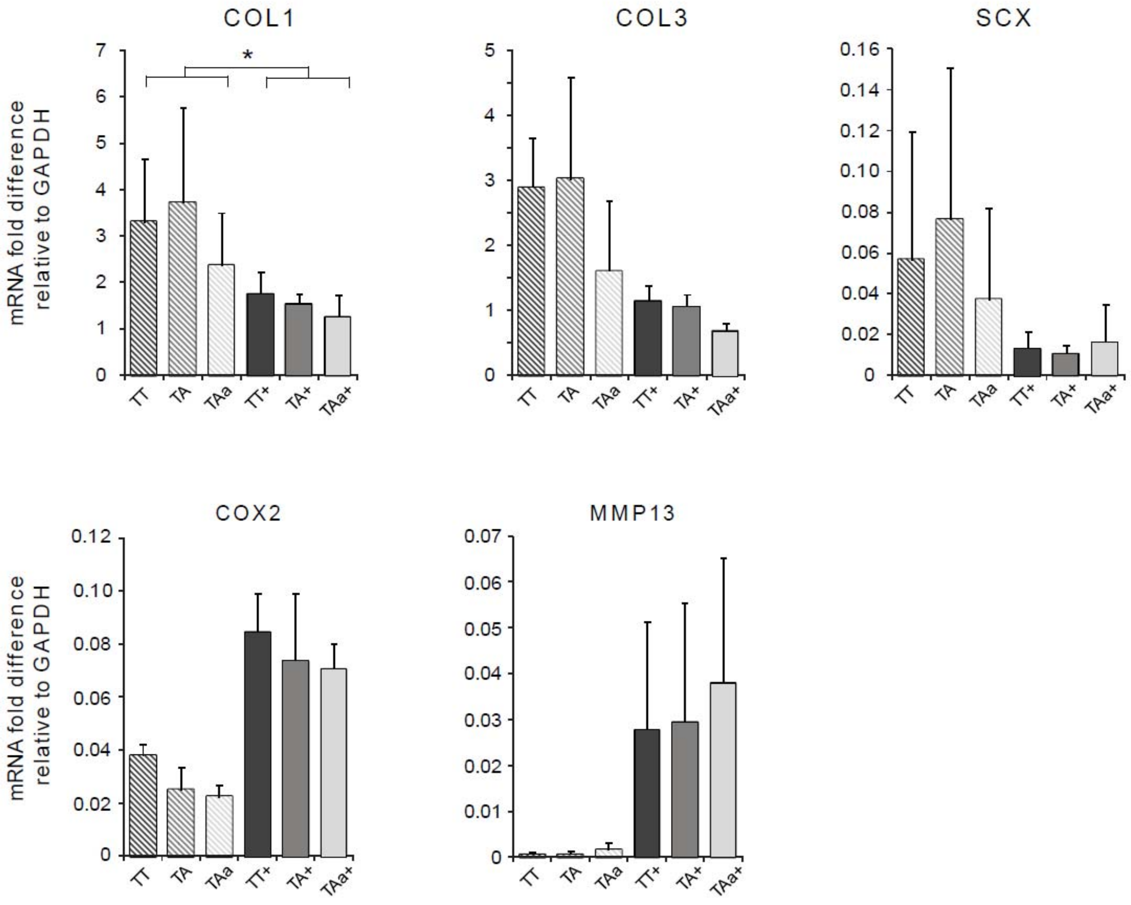

Figure S5: ASCs failed to suppress the IL-1β-induced effects on TF gene expression. TFs were cultured alone (TT), co-cultured with naïve ASCs (TA), or co-cultured with activated ASCs (TAa), with or without IL-1β for 1 day (inclusion of IL-1β indicated with "+"). Data was normalized to the housekeeping gene GAPDH (* $p < 0.05$, based on a multi-factor ANOVA with Fisher's post-hoc tests there was a significant effects of IL-1β but no effect of ASCs, N=4). [TT: TFs cultured alone, TT+: TFs treated with IL-1β, TA: TFs co-cultured with naïve ASCs, TA+: TFs co-cultured with naïve ASCs and treated with IL-1β, TAa: TFs co-cultured with activated ASCs, TAa+: TFs co-cultured with activated ASCs and treated with IL-1β].

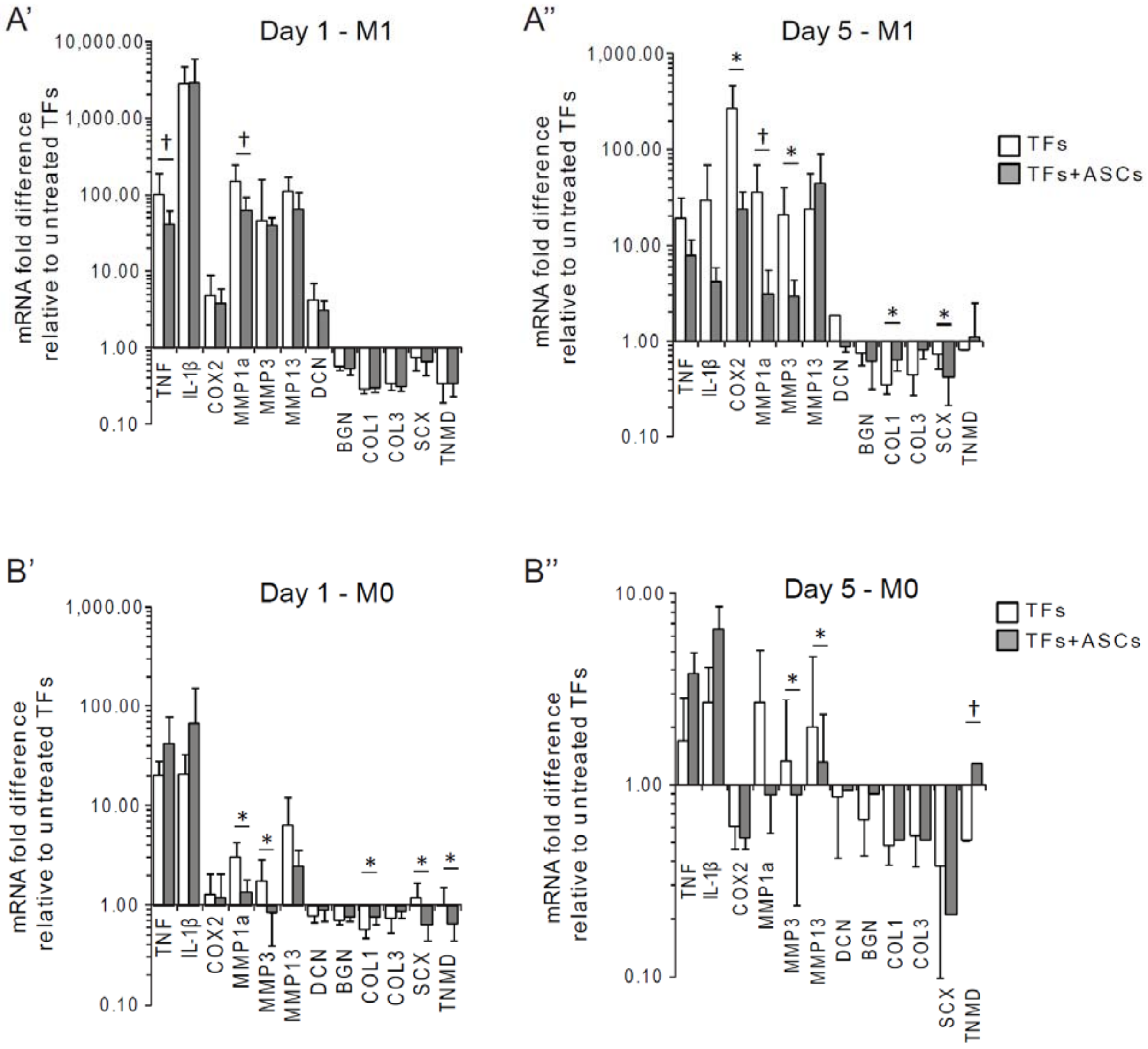

Figure S6: Changes in gene expression for TFs after culture with macrophages with and without ASCs. TFs were cultured with macrophages alone (white bars) or with macrophages and ASCs (grey bars) for 1 or 5 days. ASCs suppressed the effects of **(A)** M1 and **(B)** M0 macrophages on TF gene expression. The greatest effect of ASCs was seen for M1 macrophages on day 5 (A''). Gene expression was normalized to untreated TFs (* $p < 0.05$, N=5 for day 1, N=3 for day 5 except for bars without standard deviations, for which N=2 due to insufficient amounts of RNA for analysis).
